# Filmy Ferns (Hymenophyllaceae) and Associated Spike-Mosses (Selaginellaceae) from the Mid-Cretaceous Kachin Amber, Myanmar

**DOI:** 10.3390/biology11111629

**Published:** 2022-11-07

**Authors:** Ya Li, Yong-Dong Wang, Natalya Nosova, Ning Lu, Yuan-Yuan Xu

**Affiliations:** 1 State Key Laboratory of Palaeobiology and Stratigraphy, Nanjing Institute of Geology and Palaeontology and Center for Excellence in Life and Paleoenvironment, Chinese Academy of Sciences, Nanjing 210008, China; 2 Komarov Botanical Institute of the Russian Academy of Sciences, Prof. Popova Str. 2, 197376 Saint Petersburg, Russia; 3 University of Chinese Academy of Sciences, Beijing 100049, China

**Keywords:** ferns, fossils, *Hymenophyllites*, *Selaginella*

## Abstract

**Simple Summary:**

Three new species of filmy ferns are described in mid-Cretaceous Kachin amber, which represent the first fossil record of Hymenophyllaceae from tropical Asia. These filmy ferns and the syninclusions of spike-mosses greatly expand the diversity of the cryptogams in Kachin amber and provide additional evidence regarding the paleoenvironment. Together with other contemporaneous findings, the present fossils indicate that Hymenophyllaceae have already accumulated some notable diversity in the Cretaceous.

**Abstract:**

Filmy ferns (Hymenophyllaceae) are the most diverse lineage of the early-diverging leptosporangiate ferns with ca. 430 species widely distributed around the world but with the highest diversity in the humid tropics. However, their fossil record is scarce because of the low preservation potential of the delicate, membranous laminae. So far, no Hymenophyllaceae fossils have been reported from tropical Asia. Here, we describe some fern remains and their syninclusions (spike-mosses) in four pieces of Kachin amber from the mid-Cretaceous of Hukawng Valley, Northern Myanmar, as *Hymenophyllites angustus* sp. nov., *H*. *kachinensis* sp. nov., *H*. *setosus* sp. nov. (Hymenophyllaceae) and *Selaginella alata* sp. nov. (Selaginellaceae), respectively. These fern remains are assigned to Hymenophyllaceae based on the filmy, one-cell thick, decompound pinnatifid laminae and dichotomous venation. They represent the first fossil record of Hymenophyllaceae in tropical Asia. The growth habits of these ferns and associated spike-mosses and their implication for paleoenvironment are discussed. Our study expands the diversity of the cryptogams in mid-Cretaceous Kachin amber. Together with other contemporaneous findings, the present fossils indicate that Hymenophyllaceae have already accumulated some notable diversity in the Cretaceous.

## 1. Introduction

Molecular phylogenetic analyses reveal that during the Cretaceous Terrestrial Revolution (KTR) period around 125–80 Ma, the radiations of many lineages of land plants, including polypod ferns, epiphytic leafy liverworts and mosses, coincide with the rapid rise of angiosperms [1,2,3,4]. Recent palaeobotanical findings indicate that the mid-Cretaceous Kachin amber deposit of Myanmar is an important source of Mesozoic cryptogams and angiosperms [5,6,7], which could provide direct evidence for the above hypothesis. Indeed, a large number of cryptogamic plant inclusions has already been described from Kachin amber, including liverworts [7,8,9,10,11,12,13,14,15,16,17,18,19], mosses [20,21,22], lycophytes [23,24,25] and ferns [26,27,28,29,30,31,32,33,34,35,36]. As far as ferns are concerned, nine genera and ten species have been reported, most of which could be assigned to the extant families, including Cystodiaceae, Pteridaceae, Dennstaedtiaceae, Marsileaceae, Lindsaeaceae and Thyrsopteridaceae. Here, we recognized Hymenophyllaceae for the first time in the mid-Cretaceous Kachin amber.

The filmy ferns (Hymenophyllaceae) are an early-diverging lineage of leptosporangiate ferns [37,38,39,40], including ca. 430 extant species [39] characterized by their thin filmy lamina usually one-cell thick between veins, pubescent rhizome and marginal indusiate sori [41,42,43,44,45]. Hymenophyllaceae occur predominantly in mossy forest in tropical mountains [45,46]. Traditionally, only two genera have been recognized in this family, viz., *Hymenophyllum* Sm. with bivalved involucres (indusia) and included receptacles and *Trichomanes* L. with tubular involucres and long and projecting receptacles [47,48]. Taking into account past studies, as well as recent investigations including molecular data, Ebihara et al. [42] proposed a modern scheme of classification for this family. They divide the Hymenophyllaceae into two subfamilies (clades): the subfamily Hymenophylloideae Burnett (hymenophylloids) with a single genus *Hymenophyllum*, and the subfamily Trichomanoideae C. Presl (trichomanoids) with eight genera, including *Abrodictyum* C. Presl, *Callistopteris* Copel., *Cephalomanes* C. Presl, *Crepidomanes* C. Presl, *Didymoglossum* Desv., *Polyphlebium* Copel., *Trichomanes* L. and *Vandenboschia* Copel. [39,42]. This scheme has been widely accepted by recent researchers.

Numerous Paleozoic and Mesozoic Hymenophyllaceae-like fossils were assigned to the extinct genera *Hymenophyllites*, *Trichomanides* and *Trichomanites* particularly by 19th century researchers, with references compiled in [49,50]. However, most of these fossils lack definite evidence of a membranous habit or a marginal indusiate soral morphology [51,52]. Among them, only two species were thought to be putative members of Hymenophyllaceae [53], namely *Hymenophyllites quadridactylites* (Gutbier) Kidston from the Late Carboniferous of France [54] and *Trichomanides laxum* Tenison-Woods from the Jurassic of Queensland, Australia [55]. However, there is no convincing evidence for the presence of a tubular or bivalved indusium in *Hymenophyllites quadridactylites* [56], although this species has clusters of sporangia at the ends of the pinnule lobes, resembling extant *Hymenophyllum*. The type of material of *Trichomanides laxum* was re-examined and determined to be actually a poorly preserved leaf of the corystospermalean seed fern *Dicroidium* [51].

The oldest unequivocal representative of the Hymenophyllaceae is *Hopetedia praetermissa* Axsmith et al. from the Late Triassic (Carnian) Pekin Formation of North Carolina, USA [51]. This species is represented by creeping rhizomes, fertile tripinnate membranaceous fronds, marginal funnel-shaped indusia (involucres) and sporangia borne on a short receptacle [51]. The funnel-shaped indusia resemble those of extant genera of the subfamily Trichomanoideae, while the short receptacles are similar to those of extant *Hymenophyllum* [51]. Other convincing Hymenophyllaceae fossils do not appear until the Early Cretaceous, including *Hymenophyllites macrosporangiatus* Vachrameev from the Albian of Kazakhstan [57], *Eogonocormus cretaceum* Deng and *E*. *linearifolium* (Deng) Deng from Houlinhe Basin, Inner Mongolia, China [58,59], and *Hymenophyllum iwatsukii* Herrera et al. from Mongolia [60]. Reinvestigation of the Early Cretaceous *Acrostichopteris* Fontaine previously assigned to the Schizaeaceae by Reed [61] indicates that it could be reassigned to the Hymenophyllaceae based on characteristics of the pinnules, sori, sporangia and spores [62]. Other Early Cretaceous fossils, such as *Hymenophyllites amplus* Zheng et Zhang and *Hymenophyllites hailarense* Ren et Chen from Inner Mongolia and Heilongjiang, China [63,64,65] and *Hymenophyllum priscum* Menendez from Chile [66], are less convincing [60,67]. *Hymenophyllum axsmithii* Pigg et al. from the early Eocene Okanogan Highlands, Washington, USA represents an unequivocal member of Hymenophyllaceae in the Cenozoic [67]. All other Cenozoic records of Hymenophyllaceae, such as *Hymenophyllum confusum* Lesquereux [68], are considered unconfirmed based upon a critical re-appraisal of these reported fossils [69].

Although it is clear that the filmy ferns are ancient lineages according to their phylogenetic position [3,37,38,39], the unequivocal fossil records of them are scarce [45,51,67], mainly because of the low preservation potential for the delicate, membranous lamina and the possibility that some specimens are misidentified to other ferns or even seed ferns [51,53]. In the present paper, we describe some fern remains in the mid-Cretaceous Kachin amber from Myanmar as members of Hymenophyllaceae, namely *Hymenophyllites angustus* sp. nov., *Hymenophyllites kachinensis* sp. nov. and *Hymenophyllites setosus* sp. nov., based on several exquisitely preserved lamina fragments. They represent the first fossil record of the family in tropical Asia. Associated spike-mosses are described as *Selaginella alata* sp. nov. (Selaginellaceae). The growth habits of these ferns and spike-mosses and their implication for paleoenvironments are discussed.

## 2. Materials and Methods

Burmese amber originates from several localities at Tanai in the Hukuang Valley, Kachin State (Kachin amber) [70,71], the Hti Lin (Tilin amber, uppermost Campanian ~72.1 Ma) in the Magwe Region [72] and the Pat-tar bum southeast of Khamti (Hkamti Amber, 109.7 ± 0.4 Ma), Sagain Region [73,74]. Kachin amber contains diverse plant and animal fossils [75], while no plant fossils have been reported from Hkamti amber and Tilin amber [72,73]. The age of Kachin amber is widely regarded as the late Albian–early Cenomanian, based on the evidence of the ammonite *Puzosia* Matsumoto [76] and palynomorphs [77]. U-Pb dating of zircons of Kachin amber suggests an earliest Cenomanian age (98.79 ± 0.62 Ma) for the amber-bearing horizon [78]. Concerning the recent conflicts in Myanmar, we declare that all Kachin amber pieces mentioned in this study were collected before the year 2017 and that we followed the recommendations by Haug et al. [79].

Four pieces of Kachin amber from Myanmar contain fourteen lamina fragments of Hymenophyllaceae. They were all housed at the Collection Department of Nanjing Institute of Geology and Palaeontology, Chinese Academy of Sciences, with catalog numbers PB200744–200747. The amber inclusions were studied under a ZEISS Axio Zoom.V16 microscope equipped with a high-resolution digital camera (Axiocam 512 color). Incident and transmitted light were used simultaneously for photography. All images were digitally stacked photomicrographic composites of ca. 20–50 individual focal planes using the software package ZEN 2.3 pro for a better illustration of the three-dimensional inclusions. Terminology of the descriptions of the ferns follows Axsmith et al. [51] and Liu et al. [41].

## 3. Results

Order: Hymenophyllales A.B. Frank

Family: Hymenophyllaceae Mart.

Genus: *Hymenophyllites* H.R. Goeppert

Species: *Hymenophyllites angustus* Y. Li et Y.-D. Wang, sp. nov.

Etymology: The specific epithet refers to the narrow ultimate lobes

**Figure 1:**
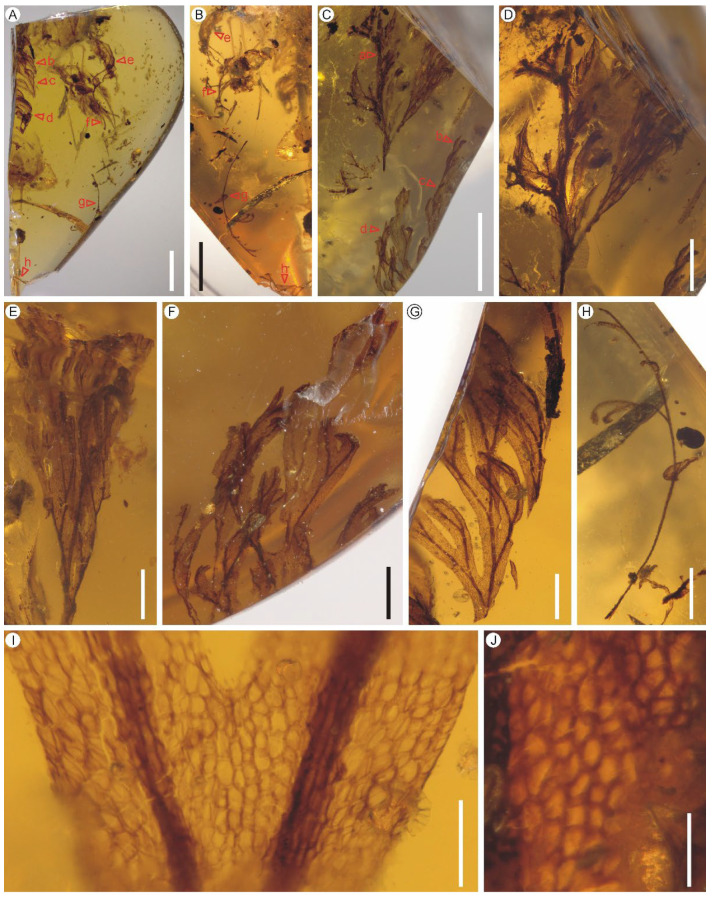
*Hymenophyllites angustus* sp. nov. from the mid-Cretaceous Kachin amber. (**A**) Top view of the amber showing seven lamina fragments. PB200744b–h. Scale bar = 5 mm. (**B**) Bottom view of the amber showing four lamina fragments. PB200744e–h. Scale bar = 5 mm. (**C**) Lateral side view of the amber showing four lamina fragments. PB200744a–d. Scale bar = 5 mm. (**D**) A tripinnatifid lamina fragment from middle portion of a lamina showing closely spaced pinnae and anadromous pinnules. Holotype PB200744a. Scale bar = 2 mm. (**E**) Portion of a pinna showing closely spaced and overlapped pinnules. Holotype PB200744a. Scale bar = 1 mm. (**F**,**G**) Pinnule fragments divided into simple or forked ultimate segments. PB200744d, c. Scale bars = 1 mm. (**H**) A young lamina fragment possibly from apical portion of a lamina. PB200744g. Scale bar = 2 mm. (**I**) Enlargement of lamina showing cell arrangement. PB200744c. Scale bar = 200 μm. (**J**) Laminar cells with straight cell walls. PB200744a. Scale bar = 100 μm.

Holotype: PB200744a (Figure 1D)

Paratypes: PB200744b–h

Type locality: Amber mines southwest of the village of Tanai ca. 105 km north of Myitkyina in Kachin State, Northern Myanmar.

Age: Late Albian–early Cenomanian, mid-Cretaceous.

Repository: Collection Department of Nanjing Institute of Geology and Paleontology, Chinese Academy of Sciences, Nanjing, China.

Specific diagnosis: Lamina fragments tripinnatifid, glabrous. Pinnae closely spaced, up to 0.8 cm long. Pinnules up to 0.5 cm long, distinctly segmented into simple or forked ultimate segments. Venation of pinnules anadromous. Segments characterized by one to several lobes. Lobes sterile, flat, narrow, slightly elongate, ca. 0.1–0.4 mm wide, entire-margined. Veins dichotomous, false veinlets absent. Cell walls straight.

Description: Eight lamina fragments are preserved in one piece of amber (Figure 1A–C). They are, at most, tripinnatifid, up to ca. 1.1–1.2 cm long, membranous, one cell layer thick between the veins, glabrous (Figure 1D–H). The rachis is winged throughout, slightly zigzag or curved, with a flat to crisped wing (Figure 1D). The pinnae are closely spaced, alternate, sessile, up to 0.8 cm long (Figure 1D). The pinnules are closely spaced, up to 0.5 cm long, often with the most proximal ones overlapping the rachis of pinna (Figure 1D). The venation of pinnules is anadromous. The pinnules are distinctly segmented into simple or forked ultimate segments which are further divided into one to several lobes (Figure 1 D–H). The lobes are sterile, flat, narrow, slightly elongated, 0.1–0.4 mm wide, entire-margined, with acute, obtuse, truncate to retuse apices (Figure 1F,G). Each lobe is vascularized by a single veinlet. Veins are dichotomous, forming a zigzag costa, and false veinlets are absent. Lamina cells are polygonal, isodiametrical to slightly elongated, 25–70 μm long and 14–48 μm wide, with straight cell walls; stomata are absent (Figure 1I,J).

Remarks: The present fern remains display the most distinctive characteristic of the family Hymenophyllaceae, viz., membranaceous, translucent laminas which are only one cell layer thick between the veins. Due to the lack of material of fertile fronds with indusia, it is really difficult to nail down the systematic relationships with the subfamilies of the Hymenophyllaceae. However, some characters allow at least the exclusion of most genera of the subfamily Trichomanoideae. For example, the present fern remains differ from *Abrodictyum* in having more than three rows of cells between midribs and laminar margins; they differ from *Cephalomanes* in having tripinnatifid lamina; they have glabrous lamina, while *Callistopteris* and *Vandenboschia* have hairs on stipes and rachises; the absence of false veinlets in the present fern remains differentiate them from most species of *Crepidomanes* and *Didymoglossum* [41,80]. Additionally, the presence of anadromous venation of pinnules also makes our fern remains more consistent with Hymenophylloideae, because most species of extant Hymenophylloideae have anadromous venation of pinnules, whereas, in Trichomanoideae, both anadromous and catadromous venation is present [60]. Due to the lack of fertile materials, we tentatively place our fern remains in the fossil genus *Hymenophyllites*, which displays great resemblance to the extant *Hymenophyllum* but still lacks some characters of it. *Hymenophyllites* represents a putative member of Hymenophyllaceae reported from the Carboniferous to Cretaceous [54,57,63,64,65].

Species: *Hymenophyllites kachinensis* Y. Li et Y.-D. Wang, sp. nov.

Etymology: The specific epithet refers to amber locality in Kachin State, Myanmar.

**Figure 2:**
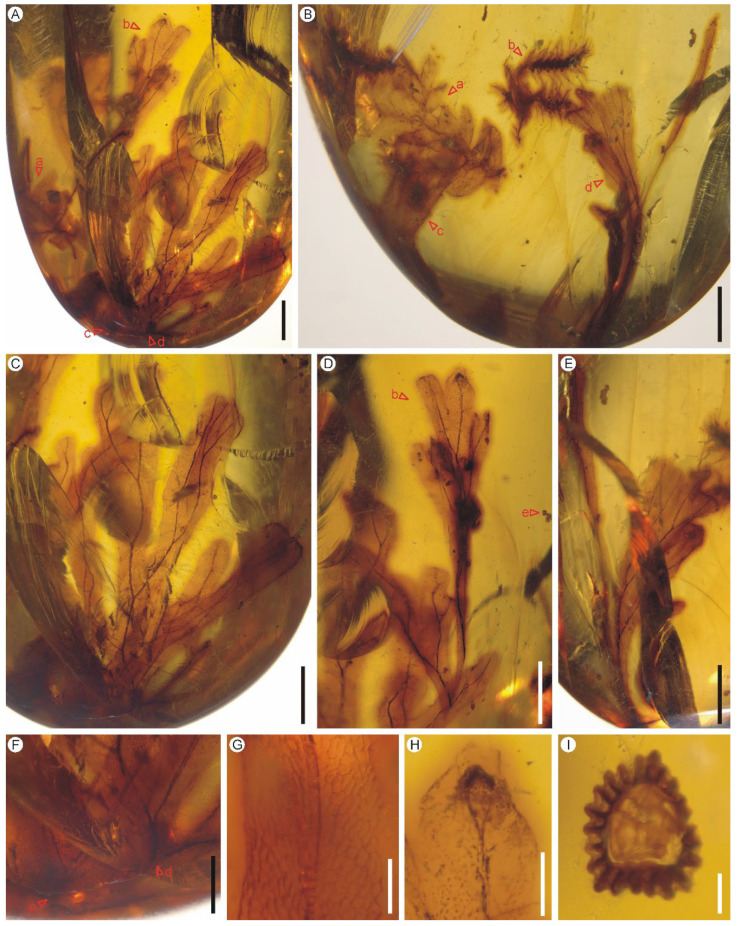
*Hymenophyllites kachinensis* sp. nov. (**A**–**H**) and its syninclusions (**I**) from the mid-Cretaceous Kachin amber. (**A**) The piece of amber containing three lamina fragments of *Hymenophyllites kachinensis* sp. nov. (PB200745b–d), as well as a sterile shoot of *Selaginella* (PB200745a). Scale bar = 2 mm. (**B**) The piece of amber containing two lamina fragments of *Hymenophyllites kachinensis* sp. nov. (PB200746c, d) and two fertile shoots of *Selaginella* (PB200746a, b). Scale bar = 2 mm. (**C**) The pinna or pinnule. Holotype PB18081914d. Scale bar = 2 mm. (**D**) A pinna or pinnule fragment and a sporangium. PB200745b,e. Scale bar = 2 mm. (**E**) Back view of the pinna or pinnule fragment. PB200746d. Scale bar = 2 mm. (**F**) Bases of two overlapped pinna or pinnule fragments. PB200745c,d. Scale bar = 1 mm. (**G**) Laminar cells with straight to slightly wavy cell walls. PB200745d. Scale bar = 300 μm. (**H**) A juvenile sorus born at apex of ultimate segment. PB200745b. Scale bar = 500 μm. (**I**) An accompanying sporangium. PB200745e. Scale bar = 50 μm.

Holotype: PB200745d (Figure 2C)

Paratypes: PB200745b,c, PB200746c,d

Type locality: Amber mines southwest of the village of Tanai ca. 105 km north of Myitkyina in Kachin State, northern Myanmar.

Age: Late Albian–early Cenomanian, mid-Cretaceous.

Repository: Collection Department of Nanjing Institute of Geology and Paleontology, Chinese Academy of Sciences, Nanjing, China.

Specific diagnosis: Pinnae or pinnules ca. 1.2–1.3 cm long, once pinnatifid, glabrous, distinctly segmented into simple or forked ultimate segments. Segments characterized by one to several lobes. Lobes sterile, flat, slightly elongate to oblong, 0.7–1.0 mm wide, entire-margined. Veins dichotomous, false veinlets absent. Cell walls straight to slightly wavy.

Description: Five lamina fragments are preserved as sterile pinnae or pinnules in two pieces of amber (Figure 2A,B). The pinnae or pinnules are ca. 1.2–1.3 cm long, once pinnatifid, membranous, one cell layer thick between the veins, glabrous, distinctly segmented into simple or forked ultimate segments which are characterized by one to several lobes (Figure 2C–F). The lobes are flat, slightly elongate to oblong, 0.7–1.0 mm wide, entire-margined with obtuse, truncate to retuse apices. Each lobe is vascularized by a single veinlet. Veins are dichotomous, forming zigzag or curved costules; false veinlets are absent. Lamina cells are polygonal, isodiametrical to elongated, 109–124 μm long and 40–76 μm wide, with straight to slightly wavy cell walls; stomata are absent (Figure 2G). A juvenile sorus is born at apex of ultimate lobe in leaf margin (Figure 2H).

Remarks: The present fern remains are similar to *Hymenophyllites angustus* sp. nov. in gross morphology, but slightly differs in having larger pinnae and/or pinnules, wider lobes of the ultimate segments and comparatively thinner veins. These characters are often used to distinguish extant species of *Hymenophyllum* [41] and thus support the present fossils as a new species of *Hymenophyllites*. The discovery of a juvenile sorus with a likely bivalved indusium born in our leaf margin indicate the present fossils may belong to the extant *Hymenophyllum* of the subfamily Hymenophylloideae. More fertile materials are still needed to finally assign them into the extant genus. Some sterile and fertile shoots of *Selaginella* (Figure 2A,B), as well as a fern sporangium (Figure 2D), are preserved as syninclusions with *Hymenophyllites kachinensis* sp. nov. The sporangium is ovate, sessile, 181 μm long and 146 μm wide, with an uninterrupted annulus (Figure 2I).

Species: *Hymenophyllites setosus* Y. Li et Y.-D. Wang, sp. nov.

Etymology: The specific epithet refers to the pinna covered with bristle-like hairs.

**Figure 3:**
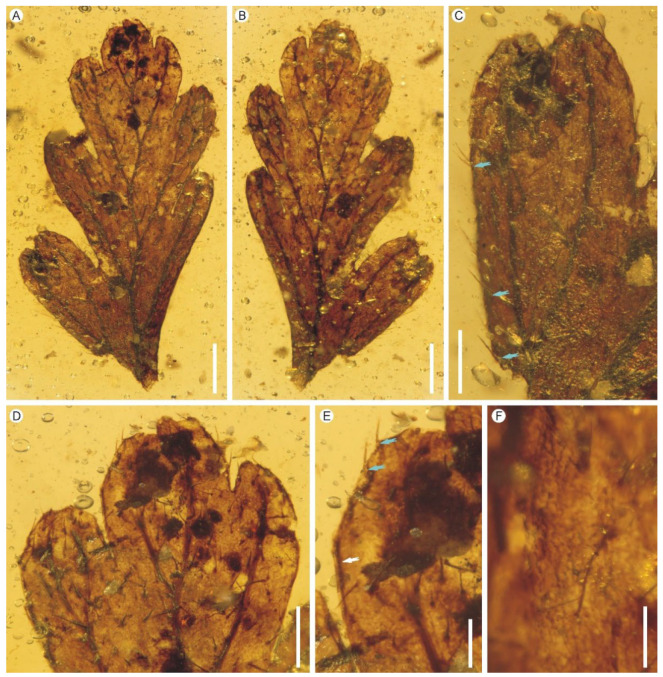
*Hymenophyllites setosus* sp. nov. from the mid-Cretaceous Kachin amber. PB200747. (**A**,**B**) Overview of the pinna or pinnule fragment in front and back views. Scale bars = 1 mm. (**C**) An ultimate segment showing the retuse apex and hairy surface with some hairs located close to the leaf margin (cyan arrows). Scale bar = 500 μm. (**D**) Enlargement of the pinna or pinnule showing obtuse to retuse apex of the segment. Scale bar = 500 μm. (**E**) Enlargement of leaf margin showing a darkened submarginal line (white arrow) and two hairs located very close to or nearly on the leaf margin (cyan arrows). Scale bar = 200 μm. (**F**) Poorly preserved laminar cells. Scale bar = 200 μm.

Holotype: PB200747 (Figure 3)

Type locality: Amber mines southwest of the village of Tanai ca. 105 km north of Myitkyina in Kachin State, northern Myanmar.

Age: Late Albian–early Cenomanian, mid-Cretaceous.

Repository: Collection Department of Nanjing Institute of Geology and Paleontology, Chinese Academy of Sciences, Nanjing, China.

Diagnosis: Pinna or pinnule once pinnatifid, membranous, probably one cell layer thick between the veins, both adaxial and abaxial surfaces sparsely covered with bristle-like hairs. Ultimate segments simple, characterized by single sterile lobes, entire-margined, 0.7–1.3 mm wide, sometimes with darkened submarginal lines. Submarginal false veinlets absent, internal false veinlets absent.

Description: The pinna or pinnule fragment is cuneate at base, once pinnatifid, 7.4 mm long, 3.2 mm wide, membranous, probably one cell layer thick between the veins, with unequally cuneate bases (Figure 3A,B). Both adaxial and abaxial surfaces are sparsely covered with bristle-like hairs (Figure 3C,D). The hairs are single, straight to curved, ca. 68–292 μm long, erect to leaning. The pinna or pinnule is distinctly segmented into 7 ultimate segments. Individual segments are simple, characterized by single sterile lobes, closely spaced, alternate, 0.7–1.3 mm wide; acroscopic and basiscopic parts are shallowly and deeply lobed, respectively; apices are obtuse, rounded to retuse; margin is generally entire but with some hairs located very close to or nearly on leaf margin (Figure 3C,E), and sometimes folded to form darkened submarginal lines (Figure 3E). Veins are dichotomous, forming a zigzag costule and seven lateral veins; lateral veins are simple or dichotomous, reaching high up to approach segment apex, ending just below apex; submarginal false veinlets are absent; internal false veinlets are absent. Lamina cells are polygonal; stomata are absent (Figure 3F).

**Figure 4:**
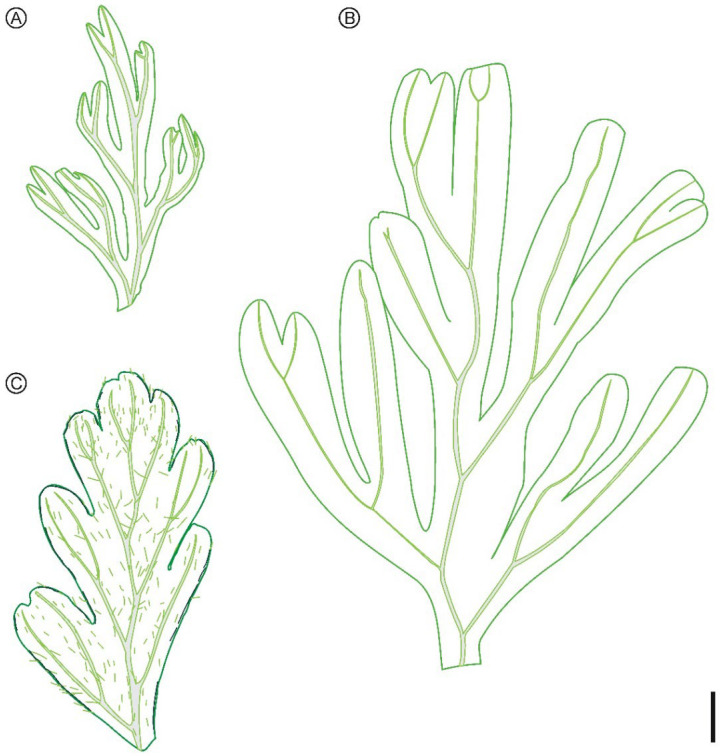
Schematic drawings of the pinnule of *Hymenophyllites angustus* sp. nov. (**A**), and the pinnae/pinnules of *Hymenophyllites kachinensis* sp. nov. (**B**) and *Hymenophyllites setosus* sp. nov. (**C**). Scale bar = 1 mm.

Remarks: The membranaceous, translucent lamina fragment indicates that the present fern remain probably belongs to the family Hymenophyllaceae. Leaf surface sparsely covered with single setose hairs is characteristic of *Hymenophyllum* subgenus *Sphaerocionium* (C. Presl) C. Chr., which is cosmopolitan with ca. 70 species, highly diversified in the Neotropics [42]. In subgenus *Sphaerocionium* today, the majority of species have stellate hairs but there are also some species with exclusively unbranched single hairs [42]. The darkened submarginal lines in our fossil look like marginal false veinlets of the extant genera *Crepidomanes* and *Didymoglossum* in the subfamily Trichomanoideae of Hymenophyllaceae [41,42,80]. But these two extant genera have glabrous leaf surface except *D*. *tahitense* (Nadeaud) Ebihara et K. Iwatsuki which has many dark brown hairs along veins [41]. The darkened submarginal lines can also be interpreted as the folds of the differentiated segment margin. Some species of *Hymenophyllum* subg. *Hymenophyllum*, e.g., *H*. *rolandi-principis* Rosenst., have a marginal seam of smaller, rounded cells that may even be darkened/indurated. Thus, the fern remain studied here closely resembles extant lineages of *Hymenophyllum* in vegetative characters and could be tentatively assigned to the fossil genus *Hymenophyllites* due to the lack of fertile characters. The subfamily assignment also awaits future confirmation when fertile material is available. The presence of bristle-like hairs, darkened submarginal lines and slightly thicker leaf lamina make the present species very easy to distinguish from the previous two new species *Hymenophyllites angustus* and *Hymenophyllites kachinensis* (Figure 4).

Order: Selaginellales Prantl

Family: Selaginellaceae Willk.

Genus: *Selaginella* P. Beauv.

Species: *Selaginella alata* Y. Li et Y.-D. Wang, sp. nov.

Etymology: The specific epithet refers to the alary keel on the abaxial side of the midrib of sporophylls.

**Figure 5:**
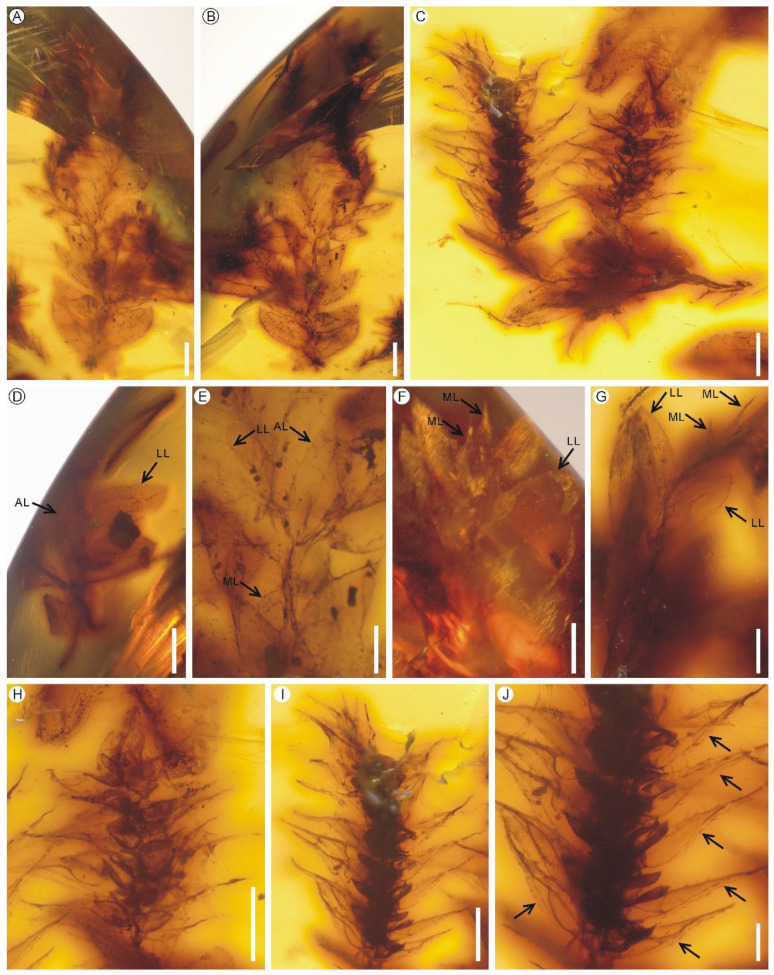
Accompanying *Selaginella alata* sp. nov. from the mid-Cretaceous Kachin amber. (**A**,**B**) A fertile shoot in dorsal and ventral views. PB200746a. Scale bars = 1 mm. (**C**) Another fertile shoot. PB200746b. Scale bar = 500 μm. (**D**) A sterile shoot showing the axillary leaf (AL). PB200745a. Scale bar = 1 mm. (**E**,**F**) Magnifications of the shoot showing the axillary, median and lateral vegetative leaves (AL, ML and LL). PB200746a. Scale bar = 500 μm. (**G**) Magnifications of the shoot showing the median and lateral vegetative leaves. PB200746b. Scale bar = 300 μm. (**H**,**I**) Strobili PB200746b. Scale bar = 500 μm. (**J**) Magnification of the strobilus (**I**) showing the alary serrulate keel (black arrows) on the abaxial side of the midrib of sporophylls. PB200746b. Scale bars = 200 μm.

Holotype: PB200746b (Figure 5C)

Paratypes: PB200745a, PB200746a

Type locality: Amber mines southwest of the village of Tanai ca. 105 km north of Myitkyina in Kachin State, northern Myanmar.

Age: Late Albian–early Cenomanian, mid-Cretaceous.

Repository: Collection Department of Nanjing Institute of Geology and Paleontology, Chinese Academy of Sciences, Nanjing, China.

Diagnosis: Shoot anisotomously branched. Vegetative leaves dimorphic, arranged in four ranks, two ranks of smaller ascending median leaves and two other ranks of larger spreading lateral leaves. Median leaves non-carinate with aristate apex and denticulate to ciliolate margin. Lateral leaves non-carinate, lanceolate, with acute to acuminate apex and entire to serrulate to ciliolate margin. Strobili born at apex of the fertile shoots, compact, tetragonal. Sporophylls arranged in four ranks, monomorphic, with long acuminate apex, dentate margin, alary serrulate keel.

Description: The two fertile shoots are 1.0 cm and 0.5 cm long and bear four and three strobili respectively (Figure 5A–C). Stems are anisotomously branched, without articulations. Rhizophores are not observed. Leaves are dimorphic, arranged in four ranks, two ranks of median leaves on dorsal or upper side of stem and branch and two other ranks of lateral leaves on lateral or lower side (Figure 5D–G). Ligules are not observed. Axillary leaves are symmetrical, lanceolate, 1.6 mm long and 0.6 mm wide (measurement of one leaf), with non-auriculate bases and ciliolate margin (with 52–81 μm long cilia) (Figure 5D,E). Median leaves are smaller, ascending, non-carinate, ovate to obovate, 0.6–0.8 mm long and 0.3–0.4 mm wide (measurement of four leaves), with aristate apex and denticulate to ciliolate margin (with 21–64 μm long teeth or cilia) (Figure 5F,G). Lateral leaves are larger and spreading, non-carinate, lanceolate, 1.2–2.0 mm long and 0.6–0.8 mm wide (measurement of eight leaves), with acute to acuminate apex and entire serrulate to ciliolate margin (with 6–88-μm-long teeth or cilia that occur mainly at the acroscopic base) (Figure 5D–G). Strobili are solitary, born at apex of main stem or branch, compact, tetragonal, 1.7–4.6 mm long and 1.4–1.8 mm wide (Figure 5H,I). Sporophylls are arranged in four ranks, monomorphic, ovate-lanceolate, 0.8–1.1 mm long and 0.1–0.2 mm high, with long acuminate apex, dentate margin (with 21–47 μm long teeth) and alary serrulate keel (with 14–32 μm long teeth) (Figure 5J). Sporophyll-pteryx is not observed. Sporangia are single per sporophyll, mature, and deeply two-valved nearly to the base. Macrospores are not observed.

Remarks: *Selaginella* is the sole extant genus of Selaginellaceae and includes approximately 750 herbaceous species, widely distributed around the world but with its highest diversity in the tropics [81,82,83,84]. *Selaginella* is an ancient lineage with fossils dating back to the Carboniferous or even Devonian [53,85]. Schmidt et al. [23,25] discovered the presence of hyperdiverse *Selaginella* in the mid-Cretaceous Kachin amber, Myanmar, including ten species of *Selaginella* subgenus *Stachygynandrum* with anisophyllous (bilateral) strobili and eleven species with isophyllous strobili. All these fossil species are erected based on the combination of a series of key characters [23,25]. Furthermore, Li et al. [24] described a new species of *Selaginella* subgenus *Stachygynandrum* with anisophyllous strobili from Kachin amber. Compared with all these *Selaginella* fossils from Kachin amber, our fossils are clearly different from the eleven species with anisophyllous strobili by having isophyllous strobili, and distinct from the two species *S*. *isophylla* A.R. Schmidt et L. Regalado and *S*. *wunderlichiana* A.R. Schmidt et L. Regalado with monomorphic and decussately arranged vegetative leaves in having dimorphic vegetative leaves arranged in four rows [25]. Our fossils resemble the rest nine species in the aspects of dimorphic vegetative leaves and isophyllous strobili, but none of the nine species has alary keel on the abaxial side of the midrib of sporophyll [25]. Alary keel is also not documented in the extant species of *Selaginella* [81,84].

## 4. Discussion

### 4.1. Comparison with Hymenophyllaceae Fossils

*Hymenophyllites angustus* sp. nov. is distinctive for its very narrow ultimate lobes (ca. 0.1–0.4 mm wide), while the lobes are more than 0.5 mm wide in other Hymenophyllaceae fossils (Table 1). *Hymenophyllites kachinensis* sp. nov. are distinguished from the Triassic *Hopetedia praetermissa* by the presence of winged costae rather than naked ones [51]. *Hymenophyllites kachinensis* has pinnatifid lamina and pinnules, whereas most coeval fossils have either fan-like lamina in *Eogonocormus cretaceum*, *E*. *linearifolium*, and *Hymenophyllites macrosporangiatus* [57,58] or flabellate pinnules in *Acrostichopteris alcainensis* Sender, *A*. *fimbriata* Knowlton, *A*. *interpinnula* Meng et Chen and *A*. *longipennis* Fontaine [62,86,87,88] (Table 1). Sterile pinnules are strongly segmented and entire-margined in *Hymenophyllites kachinensis*, while they are weakly segmented with serrate margins in *Hymenophyllum iwatsukii* [60]. The laminae are at least twice pinnatifid in *Hymenophyllites kachinensis*, but they are only once pinnate-pinnatifid in the Eocene species *Hymenophyllum axsmithii* [67]. *Hymenophyllites setosus* sp. nov. is distinctive for its leaf surface covered with single setose hairs.

Although the subfamily Trichomanoideae are a high diversified lineage with eight genera and an estimated 184 species [39], unequivocal fossils of this subfamily are extremely rare, only with *Eogonocormus* as a possible member. The thalloid fan-like form of the frond, marginal sori borne on fanlike pinnule lobes, and in situ trilete papillate spores indicate that *Eogonocormus* is close to the extant genus *Gonocormus* Bosch [58]. At present, the genus *Gonocormus* has been merged into *Crepidomanes* within the subfamily Trichomanoideae, but some members of it were transferred into *Hymenophyllum*, such as *Hymenophyllum nitidulum* (Bosch) Ebihara et K. Iwatsuki [41]. These new taxonomic treatments document how misleading morphology alone can be in this group of plants.

### 4.2. Ecological and Putatively Evolutionary Implications

It is suggested based on ancestral state reconstructions of habit that filmy ferns (Hymenophyllaceae) were ancestrally terrestrial, with epiphytism having evolved several times independently during the Cretaceous [89]. The development of more humid climates in the increasingly closed canopy forests caused by the rapid rise of angiosperms in the Cretaceous may have provided optimal conditions for epiphytic filmy ferns to diversify [89]. It is likely that *Hymenophyllites angustus* sp. nov., *Hymenophyllites kachinensis* sp. nov. and *Hymenophyllites setosus* sp. nov. were epiphytic ferns, because the extant species of *Hymenophyllum* are almost uniformly epiphytic [89]. *Hymenophyllites kachinensis* was always found together with the spike-mosses (*Selaginella*), which are understory herbaceous plants, normally growing on open soil or exposed rocks but also occasionally epiphytic in moist forests [84]. These filmy ferns and associated spike-mosses probably lived on tree trunks and therefore could easily be incased in resin.

Due to the thin filmy laminas, the extant Hymenophyllaceae predominantly live in moist mossy forest [45,90], and the evolution of the filmy ferns shows a tendency towards progressive reduction in size, which may be considered as adaptive to the extremely moist mossy zone [45]. Additionally, the Kachin *Selaginella* fossils also possess several characters suggestive of humid conditions, such as the relatively thin leaves often with fungal colonization [25]. Therefore, the discovery of the filmy ferns and associated spike-mosses from Kachin amber provides additional evidence for the presence of a tropical humid forest located close to the seashore [70,76,91] and with a diverse fern flora in the mid-Cretaceous of Kachin area. Besides the present fossils, other members of this fern flora also include one aquatic fern *Marsileaceaephyllum ciliatum* S. Wang et al. (Marsileaceae) [35], two tree ferns *Thyrsopteris cretacea* C.X. Li et R.C. Moran and *T*. *cyathindusia* Shi et al. (Thyrsopteridaceae) [33,34] and eight polypod ferns as follows: *Cladarastega burmanica* G. Poinar Jr. (Dennstaedtiaceae), *Cretacifilix fungiformis* G.O. Poinar Jr. et R. Buckley (family incertae sedis), *Cystodium sorbifolioides* L. Regalado et al. (Cystodiaceae), *Heinrichsia cheilanthoides* L. Regalado et al. (Pteridaceae), *Holttumopteris burmensis* L. Regalado et al. (family incertae sedis), *Krameropteris resinatus* H. Schneid. et al. (Dennstaedtiaceae), *Proodontosoria myanmarensis* Li et Moran (Lindsaeaceae) and an indeterminate species of Lindsaeaceae [26,27,28,29,30,31,32,36].

Divergence time estimates based on molecular data suggest that the stem group of Hymenophyllaceae may have diverged from other leptosporangiate ferns as early as the Carboniferous to Triassic [3,37,38,89,92,93], with the divergence between Trichomanoideae and Hymenophylloideae occurring during the Middle Jurassic [89]. Based on the analysis of rbcL sequence data, the family Hymenophyllaceae probably arose and first diverged in the Paleotropics, possibly in Asia [90] and subsequently dispersed from there [45]. However, prior to this study, Hymenophyllaceae fossils have never been reported from modern tropical Asia. The rarity of fossils found in tropical regions today is because of the high rate of chemical weathering under present per humid tropical conditions that dissolves any calcium or carbon-based fossils in the rocks. Amber is chemically rather inert and thus the best chance for modern tropical fossils. *Hymenophyllites angustus* sp. nov., *Hymenophyllites kachinensis* sp. nov. and *Hymenophyllites setosus* sp. nov. represent the first fossil record of Hymenophyllaceae from tropical Asia. Although much of the extant diversity of Hymenophyllaceae appears to have accumulated later in the angiosperm-dominated forests of the Cenozoic [89,94], the present fossils, along with other Cretaceous fossil records [57,58,60], indicate that Hymenophyllaceae have already accumulated some notable diversity in the Cretaceous. The study of Dubuisson et al. [93] supported the Early Cretaceous as a critical time for early diversification in *Hymenophyllum*.

## 5. Conclusions

Here, we describe fern remains from the mid-Cretaceous Kachin amber, Myanmar as three new species of *Hymenophyllites* (Hymenophyllaceae), namely *H*. *angustus* sp. nov., *H*. *kachinensis* sp. nov. and *H*. *setosus* sp. nov., and also describe associated *Selaginella alata* sp. nov. (Selaginellaceae) embedded together with *Hymenophyllites kachinensis*. The presence of these filmy ferns and associated spike-mosses are suggestive of high humidity in the source forests of this amber. These fern remains of Hymenophyllaceae represent the first fossil record of the family in tropical Asia. Our results greatly expand the known diversity of the fern flora in Kachin amber, Myanmar. Together with other contemporaneous findings, the present fossils indicate that Hymenophyllaceae have already accumulated some notable diversity in the Cretaceous.

## Figures and Tables

**Table 1:** Comparison of the present fossils with related Hymenophyllaceae fossils.

Taxon	Lamina Shape	Lamina Surface	Pinnule Shape	Lobe Width (mm)	False Veinlets	Age	Locality	Reference
*Acrostichopteris alcainensis* Sender	Pinnate to pinnate-pinnatifid	?	Flabellate	?	?	Early Cretaceous	Alcaine, Teruel Province, Spain	[62]
*Acrostichopteris fimbriata* Knowlton	Pinnate	?	Flabellate	?	?	Early Cretaceous	Montana, USA	[87]
*Acrostichopteris interpinnula* Meng et Chen	Tripinnate	?	Flabellate	0.5	?	Early Cretaceous	Fuxin Basin, Liaoning, China	[86]
*Acrostichopteris longipennis* Fontaine	Pinnate-pinnatifid	?	Flabellate	?	?	Early Cretaceous	Potomac Group, Baltimore, Maryland and Virginia, USA	[62,88]
*Eogonocormus cretaceum* Deng	Fan-like	?	–	1.0	?	Early Cretaceous	Huolinhe Basin, Inner Mongolia, China	[58]
*Eogonocormus linearifolius* Deng	Fan-like	?	–	1.0	?	Early Cretaceous	Huolinhe Basin, Inner Mongolia, China	[58]
*Hopetedia praetermissa* Axsmith et al.	Tripinnate	?	Pinnate to deeply pinnatifid	1.0	?	Late Triassic	North Carolina, USA	[51]
*Hymenophyllites angustus* sp. nov.	Tripinnatifid	Glabrous	Deeply pinnatifid	0.1–0.4	Absent	Mid-Cretaceous	Kachin amber, Myanmar	This paper
*Hymenophyllites kachinensis* sp. nov.	Pinnatifid	Glabrous	Deeply pinnatifid	0.7–1.0	Absent	Mid-Cretaceous	Kachin amber, Myanmar	This paper
*Hymenophyllites macrosporangiatus* Vachrameev	Fan-like	?	–	?	?	Early Cretaceous	Kazakhstan	[57]
*Hymenophyllites setosus* sp. nov.	Pinnatifid	With bristle-like hairs	Pinnatifid	0.7–1.3	Absent	Mid-Cretaceous	Kachin amber, Myanmar	This paper
*Hymenophyllum axsmithii* Pigg et al.	Once pinnate-pinnatifid	?	–	0.5	?	Early Eocene	Boot Hill, Washington, USA	[67]
*Hymenophyllum iwatsukii* Herrera et al.	Bipinnate	Probably with trichomes	Shallowly pinnatifid	?	?	Early Cretaceous	Tevshiin Govi and Tugrug, Mongolia	[60]

? means unknown character, and – stands for inapplicable character.

## Data Availability

All data dealing with this study are reported in the paper.
